# A formal approach to the hierarchical structures of microbial communities with negative interactions

**DOI:** 10.1016/j.csbj.2025.11.036

**Published:** 2025-11-21

**Authors:** Beatrice Ruth, Bashar Ibrahim, Peter Dittrich

**Affiliations:** a Department of Mathematics and Computer Science, Friedrich Schiller University Jena, Fürstengraben 1, Jena, 07743, Germany; b Department of Mathematics & Natural Sciences and Centre for Applied Mathematics & Bioinformatics, Gulf University for Science and Technology, Hawally, 32093, Kuwait; c European Virus Bioinformatics Center, Leutragraben 1, Jena, 07743, Germany

**Keywords:** Microbial communities, Persistent subspace, Chemical organization theory, Negative interactions, Inhibitory resource

## Abstract

Microbial communities typically consist of numerous species that coexist through intricate mutual dependencies. Understanding the structure of these communities and the interactions among their species is essential for explaining their functions and predicting their behavior. In this study, we follow the idea that a community organizes itself into a hierarchy of potentially persistent sub-communities. Previously, this hierarchy was described using Chemical Organization Theory (COT). However, that approach did not account for negative interactions. Here, we enhance the theory by incorporating negative interactions through an inhibitory resource called a toxin. For simplicity, we assume that a taxon sensitive to a toxin cannot coexist with a taxon that produces that toxin. Our results demonstrate that introducing a toxin reduces the number of organizations, with the extent of this reduction depending on various modeling parameters. Further, we show that the usage of essential resources leads to a computationally NP-hard transformation problem into direct taxa interactions. Additionally, we demonstrate that the number of measurements required to infer all persistent subspaces increases. We determine which groups of species are mutually excluded due to toxin interactions. Besides toxic interactions, it is also possible to infer cross-feeding aspects of the microbial community, for which a potential algorithm is outlined and illustrated by an example.

## Highlights

• In this manuscript, we enhance the COT framework by integrating toxin-based negative interactions to model microbial community dynamics.
• The introduction of toxins leads to a reduction in the number of persistent sub-communities, with the extent of this reduction varying based on model parameters.
• We quantify the increase in measurements required to infer all persistent subspaces and identify the minimal number of toxins needed to explain the observed interactions.
• The study also highlights how the introduction of toxins impacts the variety of measurements and the need for additional experiments to accurately characterize the community’s structure.

## Graphical Abstract

**Figure:**
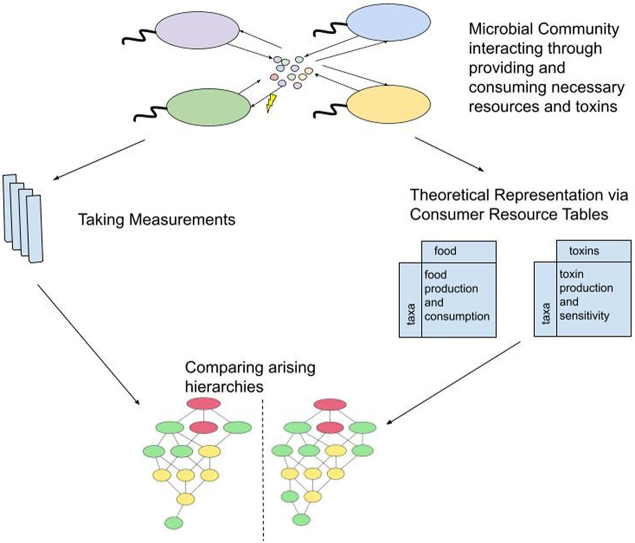

## 1 Introduction

Research into microbial communities, which are found nearly everywhere on our planet [2], [33], continues to uncover new insights into their diverse influences [5], [11], [35]. Directly referencing humans, it is evident that the composition of our intestinal microbiome has a decisive impact on our health [9], [26], [29]. Moreover, bacterial communities in aquatic and terrestrial environments play a crucial role in biogeochemical cycles [2], [5], [11], [35].

Time series measurements of these bacterial communities reveal a complex, multi-layered network with varying compositions [24], [25], [27], [32], [34], [35], [36]. From an evolutionary perspective, such diversity is puzzling: classical theory would predict competitive exclusion, unless highly specific coexistence conditions are met [4], [7], [13]. These conditions go beyond resource abundance, often involving indirect interactions such as facilitation and inhibition via metabolic products or toxins [8], [17], [28].

Mechanistic consumer-resource models provide a framework to explain these complex interactions [15], [19]. In particular, the Black Queen Hypothesis posits that species may survive by outsourcing costly metabolic functions, relying on shared byproducts [20]. Such dependencies can stabilize community structures and enhance diversity, provided that essential resources and byproducts are exchanged within the community.

Building on this, Chemical Organization Theory (COT) has been proposed to identify self-maintaining and closed subsets of taxa - called organizations - and to describe their interrelations through a hierarchy that reflects the structural constraints of community assembly [10], [25].

However, microbial communities also include negative interactions, such as inhibition via secreted toxins. In this study, we extend the COT framework by introducing such negative interactions in a simplified consumer-resource model. This allows us to characterize how both facilitative and inhibitory processes shape the space of stable microbial configurations. While there is evidence for microbial communities to be governed by negative interactions [7], [8], [17] there are also studies highlighting the strong impact of positive interactions [15], [16], [19], [28].

The balance between positive and negative interactions determines which groups of species can coexist in a self-sustaining manner. These stable coexistence states form the basis for a hierarchical representation of microbial community organization. The resulting hierarchy is closely linked to community assembly graphs [8], [30] without requiring dynamical knowledge. Community graphs usually contain the reachable attractors as well as their dynamical changes based on the arrival and departure of species within one environment [8], [30]. Within an assembly graph, not every organization can be identified in each environment. An assembly graph is a subset of the hierarchy of organizations. So, while an assembly graph focuses on particular build-up processes of a community, the hierarchy of organizations provides a global overall picture of all possible subspaces through which a build-up process can proceed.

In this study, we investigate how such a hierarchy can be systematically derived from a given consumer-resource model extended with negative interactions (toxins). We demonstrate that this model structure leads to a well-defined hierarchy of potentially persistent sub-communities. Based on this, we explore how observational data - such as species presence at stationary time points - can be used to reconstruct the underlying hierarchy. Finally, we discuss how this framework enables insights into possible cross-feeding relationships and the role of inhibitory interactions, such as the minimal number of toxins required to explain observed patterns of mutual exclusion.

## 2 Results

We start by introducing the model describing the microbial community. Next, we extend chemical organization theory to handle negative interactions, which in our formulation are implemented as toxin-mediated inhibition. While this explicitly refers to inhibitory toxins, the framework can be interpreted more generally as representing any type of interaction that enforces exclusive coexistence between species. Then we study the resulting hierarchical structure of organizations, the factors on which it depends, and how it is influenced by negative interactions. Finally, we investigate the measurement effort required to reconstruct the hierarchy and how the hierarchy helps to reconstruct a model (Table 1).

**Table 1:** Glossary

Term	Informal explanation	Where explained
Organization	A set of taxa that can collectively persist because all resources they need are produced within the set, and none are destroyed by toxins they are sensitive to.	Results § Consumer-resource-toxin model and § Organization with negative interactions
Hierarchy	The ordered structure showing how organizations relate to each other through inclusion; a large organization can contain a smaller one. We say that this smaller organization is below the larger organization.	Fig. 1(D) and (E), Results § The extended hierarchy
Union/Intersection	Mathematical operations describing how two organizations combine (union) or overlap (intersection).	Fig. 1(D) and (E), Results § The extended hierarchy
Interaction cluster	Those taxa of an organization that do not occur in any organization below — they form the basic “building blocks” of coexistence because they must interact.	Fig. 3, Results § Hierarchical structure governed by its interaction clusters
Measurement	A set of taxa obtained from an experimental observation (e.g., 16S rRNA), considered as a persistent community composition (an organization).	Results § Measurement effort to reconstruct the hierarchy and Methods § Simulating measurements
Neutral Encounter measurement	An experiment where each possible organization of a model or a hierarchy is equally likely to be observed, independent of starting taxa.	Results § Measurement effort to reconstruct the hierarchy and Methods § Simulating measurements
Random encounter experiment	An experiment starting with a random taxa combination, keeping the largest contained organization after toxin effects.	Results § Measurement effort to reconstruct the hierarchy and Methods § Simulating measurements

### 2.1 Consumer-resource-toxin model

**Fig. 1:**
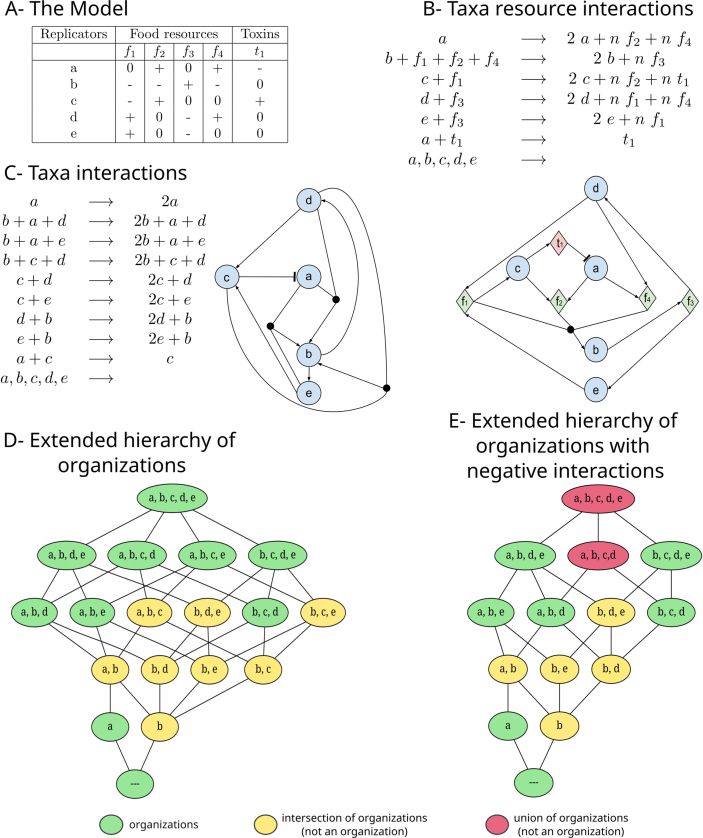
Process of deriving the hierarchical structure from a known model. (A) The model consists of five taxa (a,b,c,d,e); four food resources ($f_{1},f_{2},f_{3},f_{4}$), which can be produced (+) or consumed (−); and one toxin ($t_{1}$), which can be produced (+) or which can suppress a taxon (−). These tables can then be converted to a reaction network including taxa and resources (B) or to a network of taxa interacting directly, not including any resources (C). If interactions are based on resources the stoichiometric coefficients for resource production n should be sufficiently high to compensate their consumption. The extended hierarchy of organizations not considering toxins (D) and considering toxins (E) shows, in addition to organizations (green), sets that are not organizations but that can be generated as the union (red) or intersection (yellow) of organizations. (For interpretation of the references to colour in this figure legend, the reader is referred to the web version of this article.)

Like in the consumer-resource model introduced by MacArthur [18], in our model, taxa interact solely indirectly via resources. However, in our model we focus exclusively on essential resources that directly contribute to internal community interactions. Furthermore, resources are simplified as either being consumed or produced by a taxon (Fig. 1(A)). Thus, we abstract quantitative amounts and quantitative kinetic rates. This reduction allows us to ignore external resources - that is, those not produced within the community - as they do not influence the structural analysis at the level of abstraction considered here. As a result, the model focuses on resources relevant for cross-feeding and on strong negative interactions (toxins) that exclude coexistence, as these are the key drivers of community organization in our framework [7], [12], [13]. Additional external resources or weaker interactions, however, may influence which of the predicted organizations can actually be realized in natural systems.

To represent negative interactions within the community, we introduce inhibitory resources, which we refer to as toxins. These toxins serve as a general abstraction for various biologically relevant coexistence-excluding mechanisms, including amensalism, predator–prey relationships, and competition. What these interactions share is that they prevent stable coexistence between the involved taxa in the absence of a mediating third party. In our model, we formalize this by assuming that if one taxon produces a toxin to which another is sensitive, both taxa cannot be part of the same self-sustaining organization. This ensures that the interaction leads to mutual exclusion, meaning that a stable coexistence of the two taxa is impossible within a closed system. The toxin thus represents not just chemical inhibition, but any mechanism that enforces such exclusive outcomes.

**Definition (consumer-resource-toxin model):** Let S∈N be the number of taxa, M∈N the number of food resources, and T∈N the number of toxins. A consumer-resource-toxin model M is a tuple (S,M,T,R,T)=M, which contains a food resource table $R\in {\left\{-1,0,1\right\}}^{S\times M}$ and a toxin table $T\in {\left\{-1,0,1\right\}}^{S\times T}$ such that both tables contain the same S taxa in the same ordering.

**Definition (food resource table):** A food resource table is a matrix $R=(\rho_{i,\alpha })\in {\left\{-1,0,1\right\}}^{S\times M}$ representing the production and consumption of M food resources by S taxa such that (1) the production of a resource α∈{1,…,M} by a taxon i∈{1,…,S} is represented by $\rho_{i,\alpha }=1$, (2) a taxon i being dependent on a resource α is represented by $\rho_{i,\alpha }=-1$, while (3) the remaining entries are zero.

**Definition (toxin table):** A toxin table is a matrix $T=(\tau_{i,\beta })\in {\left\{-1,0,1\right\}}^{S\times T}$ representing the production of T toxins and their effects on S taxa such that (1) the production of a toxin β∈{1,…,T} by a taxon i∈{1,…,S} is represented by $\tau_{i,\beta }=1$, (2) a taxon i affected by a toxin β is represented by $\tau_{i,\beta }=-1$, while (3) the remaining entries are zero.

Given a model M, for each taxon i the set of required food resources $\delta_{i}=\{\alpha |\;\rho_{i\alpha }=-1\}$, the set of produced food resources $\epsilon_{i}=\{\alpha |\;\rho_{i\alpha }=1\}$, the set of produced toxins $\zeta_{i}=\{\beta |\;\tau_{i\beta }=1\}$, and the set of effective toxins $\eta_{i}=\{\beta |\;\tau_{i\beta }=-1\}$ is defined.

As exemplified by Fig. 1(A), a model can be fully described by a compact table. In this example there are S=5 taxa {a,b,c,d,e}, M=4 food resources $\left\{f_{1},f_{2},f_{3},f_{4}\right\}$, and T=1 toxin $\left\{t_{1}\right\}$. For example, taxon b requires food resources $\delta_{b}=\{f_{1},f_{2},f_{4}\}$ to grow and produces food resources $\epsilon_{b}=\{f_{3}\}$. Taxon a does not require any food resource ($\delta_{a}=\emptyset$), produces food resources $\epsilon_{a}=\{f_{2},f_{4}\}$, and is affected by toxin $\eta_{a}=\{t_{1}\}$, which is produced by taxon c ($t_{1}\in \zeta_{c}$). A visualization can include resources and toxins (Fig. 1(B)) or omit them (Fig. 1(C)).

Without loss of generality we assume that a taxon needs all its required food resources to grow and cannot grow if any toxin to which it is sensitive to is produced by the community.

To make a conceptual link to COT, the model can be represented as a reaction network derived from a food table and a toxin table. In this reaction network, taxa act as replicators that consume required food molecules and may produce new resources or toxins. For example, $b+f_{1}+f_{2}+f_{4}⟶2\;b+n\;f_{3}$ shows that taxon b consumes its required food resources $\delta_{b}=\{f_{1},f_{2},f_{4}\}$ and produces metabolite $f_{3}$.

Similarly, a taxon may produce toxins while replicating, e.g., $c+f_{1}⟶2\;c+n\;f_{2}+n\;t_{1}$

where $t_{1}$ inhibits sensitive taxa. The corresponding inhibition reaction is represented as $a+t_{1}⟶t_{1}$, meaning that taxon a is eliminated in the presence of toxin $t_{1}$.

Finally, spontaneous death is captured by a,b,c,d,e⟶. All stoichiometric coefficients are set to one on the educt side and to sufficiently high n on the product side, as the model focuses on the structural topology rather than quantitative stoichiometry.

Alternatively, the interaction network can be represented without explicitly including resources and toxins, as shown in Fig. 1(C). However, the relationship between the full reaction network (including resources and toxins) and this reduced, taxa-only network is far from straightforward. In fact, computing such a reduced network is computationally NP-hard, as it involves identifying, for each taxon, a minimal set of other taxa that together produce all the resources it requires. This corresponds to the well-known minimal hitting set problem (see Methods § Transformation of a model to a reaction network).

Despite this complexity, applying Chemical Organization Theory (COT) to either the full or the reduced network leads to the same set of organizations, since both representations capture the same underlying dependencies between taxa.

### 2.2 Organization with negative interactions

In chemical organization theory (COT) [10], a chemical organization with respect to a given reaction network is a closed and self-maintaining set of species. An organization distinguishes a set of species that can be persistent from those sets of species that cannot be persistent [10], [23]. Thus, the hierarchy of organizations (Hasse diagram) provides a complete overview of all possible subspaces (sets of species) of a dynamical system governed by the given reaction network.

Analogously to a chemical organization we here refer to an organization as a closed and self-maintaining set of taxa. Additionally, we will consider negative interactions caused by the toxins. Instead of defining an organization with respect to a reaction network, we define an organization by referring directly to the tables of the consumer-resource-toxin model. This is equivalent to defining an organization with respect to a reaction network that is derived from the model, cf. Fig. 1(D).

The two key properties defining an organization being closure and self-maintenance have to be accounted for as in the following. Closure means that the taxa within a set cannot give rise to any taxon outside the set. In our case, this property is trivially satisfied for any subset of taxa, since taxa are considered as self-replicators that do not generate new taxa. Self-maintenance requires that each taxon in the set can persist using only the resources produced by other members of the set. Since we assume a general decay of each taxon and do not model detailed stoichiometry or kinetics, this condition reduces to the requirement that for every resource a taxon needs, there exists another taxon in the set that produces it.

Based on these assumptions, an organization within the model, not considering toxins yet, can be interpreted as: a set of taxa O⊆{1,…,S} such that every required resource is produced within the community (formally: for each taxon $i\in O\wedge \forall \alpha \in \delta_{i}\exists j\in O,\alpha \in \epsilon_{j}$).

The set of taxa forming an organization O⊆{1,…,S} has to satisfy the condition, that for each taxon i∈O and for every resource $\alpha \in \delta_{i}$ required by taxon i, there exists at least one taxon j∈O such that taxon j produces resource α ($\alpha \in \epsilon_{j}$).

To also consider negative interactions, we make the following assumption: Each toxin acts at a much higher rate than any possible replication, resulting in the extinction of all taxa affected by the corresponding toxin. Furthermore, a toxin cannot be neutralized. Hence, a set of taxa can only form an organization, if none of its members are affected by any toxin produced within the set of taxa. This leads to the following definition:

**Definition (organization):** Given a model M=(S,M,T,R,T), an organization O⊆{1,…,S} is a set of taxa such that (1) every consumed metabolite is produced within the organization (formally: $\forall i\in O\wedge \forall f\in \delta_{i}\;\exists j\in O,f\in \epsilon_{j}$); and (2) no member of the organization is affected by any toxin produced within it(formally: $\forall i\in O\wedge \forall t\in \eta_{i}\;∄j\in O,t\in \zeta_{j}$). Condition (1) ensures metabolic maintenance as in the scenario without toxins, while condition (2) introduces ecological compatibility: the set must not contain self-destructive interactions, i.e., no taxon can be eliminated by toxins originating from within the organization itself. Together, these conditions extend the notion of an organization from purely cooperative systems to systems that also include antagonistic or inhibitory interactions.

However, when toxins are introduced, some combinations of taxa that were previously compatible now become infeasible, and the resulting set of organizations may lose the lattice property. That is, the join (union) of two organizations may no longer itself be an organization.

**Definition (non-persistent union (intersection)):** A set of taxa A⊆{1,…,S} is a non-persistent union (non-persistent intersection), if it is a union (intersection) of two organizations, but is not itself an organization.

Non-persistent unions (and intersections) represent transient or unstable combinations of organizations — sets that can momentarily arise from the coexistence of smaller stable organizations but cannot maintain themselves due to internal incompatibilities (e.g., toxin effects).

Including these non-persistent sets in the structure restores the lattice property of the organizational hierarchy, such that any two organizations have a well-defined union and intersection.

It has been shown that there is a proven link between organizations and non-spatial [10], [22] and spatial dynamics [14], [23]. Only taxa (or molecules, in general) that form an organization can be persistent in the corresponding dynamical system [23]. Consequently, it can be assumed that the sets of taxa observed as stable or recurring in experiments most likely correspond to organizations in the underlying chemical or ecological model.

### 2.3 The extended hierarchy

**Fig. 2:**
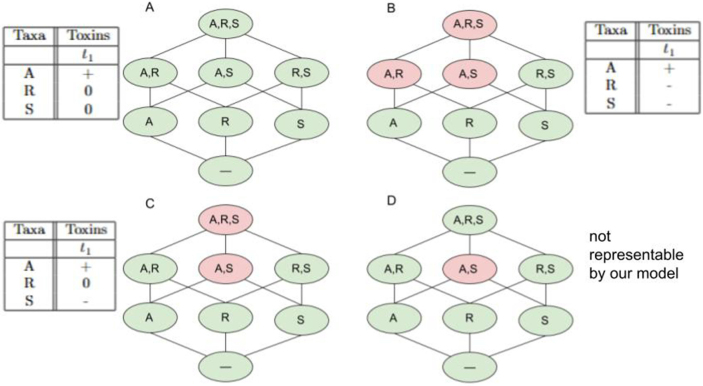
Extended hierarchies of four different instances of the three-taxa BARS model, in which taxon A produces a toxin affecting taxa S and R, while R can be resistant. The four instances differ in the strength of the inhibitory effect. (A) Without sufficient inhibition strength, A cannot outcompete S; consequently, all combinations are persistent and can coexist. (B) With very strong inhibition, combinations containing both A and S, and even A and R, become dynamically unstable. These sets appear as non-persistent unions, while each taxon still survives in monoculture. (C) At moderate inhibition, only the pair {A,S} remains unstable, and the negative connection between A and R vanishes. (D) Under specific conditions, higher-order interactions can even neutralize the negative effect between A and S, as experimentally observed by [1]. The resulting hierarchy cannot be represented by our current model, assuming only a food resource table and a toxin table (Fig. 1(A)).

A hierarchy is illustrated by a Hasse diagram, in which organizations (shown in green) and other relevant sets are arranged from bottom to top according to the number of taxa they contain - from the smallest set at the bottom to the largest at the top. A line is drawn between two sets if and only if one is a subset of the other and there is no set in between. Importantly, along any path, a green node (organization) can never appear above a red node (non-persistent union). This follows from the assumption that a toxin cannot be neutralized in our model: a toxin that prevents a given set from being an organization (rendering a red node) would also act in any superset of that set. Consequently, there exist hierarchies of persistent subspaces that cannot be represented within the model structure investigated here (Fig. 2(D)).

The hierarchical structure of a community, visualized by this extended hierarchy, consists of organizations together with their intersections and unions, which are not necessarily organizations themselves (Fig. 1(D) and (E)).

An organization represents a combination of taxa that have the general potential to coexist and is therefore considered as a potentially observable unit in experimental or dynamical contexts (Fig. 1(D) and (E), green nodes).

The non-persistent unions (shown in red) emerge only when toxins are considered and mark boundaries of coexistence where negative interactions prevent a stable organization from forming (Fig. 1(E)). If no toxins are present, every union of organizations remains an organization (Fig. 1(D)).

The non-persistent intersections (shown in yellow) highlight shared structural or functional components between different organizations. They represent sets of taxa that are common to multiple organizations but are not independently self-sustaining. This indicates that at least one required resource is not produced within the non-persistent intersection.

### 2.4 Illustration with the BARS model

To illustrate our framework, we analyze the BARS (Bacillota A+R+S) microbial community model by Aguilar-Salinas and Olmedo-Álvarez [1], consisting of three bacterial strains (A, R, and S) with distinct inhibitory and resistance traits. Each strain can grow independently; thus, the community structure arises purely from antagonistic interactions rather than resource dependencies.

Depending on the individual interaction strengths-such as the inhibition exerted by A on S and the resistance of R-the resulting dynamical outcomes differ, leading to distinct hierarchical organizations (Fig. 2). Assuming weak inhibition, all taxa can coexist, forming a complete hierarchy. Assuming strong inhibition renders {A,S} and even {A,R} dynamically unstable, corresponding to non-persistent unions. At intermediate inhibition, only {A,S} remains unstable, while under specific conditions, higher-order effects may even neutralize this negative interaction.

This example demonstrates how the extended hierarchy framework captures and visualizes the structural outcomes of ecological interactions. It provides an abstract yet precise representation of how variations in pairwise and higher-order effects reorganize the coexistence hierarchy - linking measurable community dynamics to underlying interaction principles.

### 2.5 Hierarchical structure governed by its interaction clusters

The hierarchical structure of a community, as outlined in the previous sections, captures how organizations are related through unions and intersections. However, this representation alone does not reveal the internal structure of an organization, that is, how subsets of taxa within a given organization interact and depend on one another. To gain deeper insight into this internal level, we introduce the concept of interaction clusters, which underlie the nodes of the extended hierarchy and describe the elementary building blocks from which organizations emerge.

Interaction clusters identify groups of taxa that are functionally cohesive — connected through direct or indirect resource dependencies — and that jointly form the minimal self-contained substructures within the community. Each interaction cluster therefore represents a connected subcommunity, where the viability of one member depends on the presence of others. By resolving organizations into their interaction clusters, we can thus understand how higher-order organizations arise from the combination and expansion of these smaller interdependent groups of taxa.

Formally this internal structure can be captured by the following definition.

**Definition (interaction cluster):** An interaction cluster C of an organization $O_{i}$ ($C\subseteq O_{i}$) denotes those taxa that do not appear in any organization $O_{j}$ below $O_{i}$ ($O_{j}\subset O_{i}$).

So, an organization $O_{i}$ is the union of all organizations $O_{j}$ it contains plus its interaction cluster C, which can be empty.

This definition isolates those groups of taxa that cannot be further decomposed into smaller, self-sustaining organizations within the same branch of the hierarchy. They therefore represent the irreducible functional cores of the community structure.

We can show that all taxa within such an interaction cluster are connected, meaning that a path of food resource dependency links any pair of taxa within the cluster. To prove this by contradiction, assume that an interaction cluster could be divided into two non-empty subsets A and B, where A provides no resources for B. Then the set $O_{i}/A$ would itself be an organization contradicting the definition of an interaction cluster as consisting exclusively of taxa not present in any subset organization such as $O_{i}/A$. Hence, the taxa in each interaction cluster must form a single connected component in terms of resource dependencies.

Having established their internal connectivity, we can now distinguish between different types of interaction clusters based on whether they can maintain themselves independently or only in the presence of additional taxa. Accordingly, we refer to these two types as context-free and context-dependent interaction clusters.

**Fig. 3:**
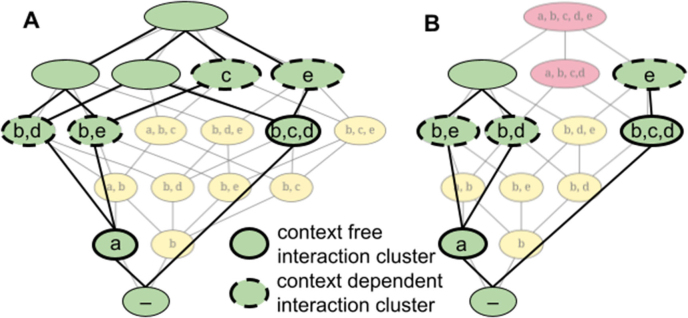
Interaction cluster within the hierarchy of organizations. Panel A highlights the interaction clusters within the organizations of Fig. 1(D) and Panel B highlights the interaction clusters within the organizations of Fig. 1(E). Context-free interaction clusters are marked with a thick solid border, and context-dependent interaction clusters are marked with a thick dashed border. An interaction cluster consists of the “newly” appearing taxa within the organization (green node). (For interpretation of the references to colour in this figure legend, the reader is referred to the web version of this article.)

A context-free interaction cluster is self-sustaining and therefore constitutes an organization itself, as all resources required by its taxa are also produced within the interaction cluster. In the example shown in (Fig. 1(D)), the organizations {a} and {b,c,d}, represent the two context-free interaction clusters of the network (Fig. 3(A)).

Context-dependent interaction clusters, in contrast, rely on the presence of additional taxa to form a complete organization. They can expand context-free clusters to generate further organizations. In the example taken here, there are four context-dependent interaction clusters, namely {b,d},{b,e},{c}, and {e}. For instance, the interaction cluster {a} can be expanded by either {b,d} or {b,e}, yielding the organizations {a,b,d} and {a,b,e}, respectively.

Each organization that introduces a new interaction cluster corresponds to one elementary organization [6]. These elementary organizations represent the minimal units of structural innovation within the hierarchy: whenever a new cluster emerges, it defines a new possible combination of taxa that can coexist. All remaining organizations in the system can then be obtained as unions of these elementary organizations, reflecting the modular buildup of the community structure.

The introduction of toxins alters this modular structure by removing certain elementary organizations and their corresponding clusters. As described in the previous section, this process reduces the overall hierarchy of organizations and limits the number of viable combinations of taxa.

While non-persistent unions of organizations are represented as red nodes within the extended hierarchy, toxin-affected elementary organizations are not directly visible in this representation. This is because an elementary organization corresponds to a single branch of the hierarchy rather than to a union of distinct branches. When an elementary organization is inhibited by a toxin, it does not produce a red node but simply vanishes from the hierarchy, along with the interaction cluster that defines it. The inhibition of an elementary organization occurs when one or more taxa within its up-building path of interaction clusters are sensitive to a toxin produced by another taxon in the same path. In such a case, the corresponding combination of taxa can no longer coexist, and the elementary organization ceases to exist. Consequently, only interaction clusters that contain, at most, either the producers of a given toxin or the taxa sensitive to that toxin remain viable (Fig. 3(B)). This selective loss reshapes the internal structure of the hierarchy by removing specific branches and reducing the number of potential pathways through which higher-level organizations can emerge.

This selective loss of elementary organizations and their associated interaction clusters reduces the combinatorial diversity of taxa assemblies within the community. As a result, the overall hierarchy becomes smaller and more constrained: certain organizations and their intersections can no longer form, limiting the number of viable community structures (Fig. 1(E)). In this way, toxin-mediated inhibition directly shapes both the internal composition of organizations and the global patterns of coexistence in the hierarchy. In the illustrated example, taxon c produces a toxin to which a is sensitive, thereby affecting the three organizations {a,b,c,d}, {a,b,c,e} and {a,b,c,d,e}. While {a,b,c,d} and {a,b,c,d,e} persist as red nodes, the elementary organization {a,b,c,e} disappears. Consequently the three intersections {a,b,c},{a,b,e} and {b,c} (yellow nodes) also vanish - even though only one of them contains both the toxin producer c and the sensitive taxon a.

### 2.6 Factors influencing the number of organizations

The maximal number of possible organizations is given by the power set of the taxa set under consideration. For example, a model with S=10 taxa can have up to 1024 different organizations. However, not all combinations of taxa are viable: only those sets that produce all their individually required resources qualify as organizations.

**Fig. 4:**
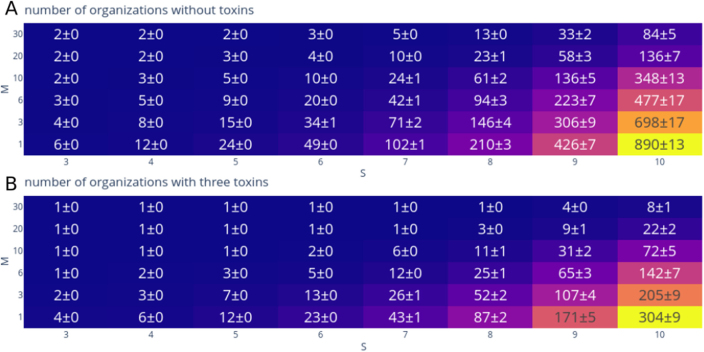
Comparison of the number of organizations, depending on the number of food resources M, each having $p_{\alpha ,\text{produce}}=0.\overset{―}{3}$ and $p_{\alpha ,\text{require}}=0.\overset{―}{3}$, and number of taxa S, without toxins (A) and with three toxins (B), each having $p_{\beta ,\text{produce}}=0.1$ and $p_{\beta ,\text{sensitive}}=0.1$. The number of organizations decreases with the uptake of resources while it increases when more taxa are added to the system. The presence of toxins drastically decreases the number of possible organizations within a community. Depicted is the mean and the standard error of the mean based on 100 randomly generated models.

To investigate how resource diversity affects organization formation, Fig. 4(A) shows results for randomly generated models. In these models, each food resource α is produced with probability $p_{\alpha ,\text{produce}}=0.\overset{―}{3}$ and required with probability $p_{\alpha ,\text{require}}=0.\overset{―}{3}$. As the total number of resources M increases, specialization in both production and consumption rises: each taxon tends to depend on a subset of resources produced by multiple different taxa rather than a single individual taxon. Consequently, small organizations become less frequent, and the extended hierarchy contains an increasing number of non-persistent intersections of organizations (data not shown).

Next we consider the influence of inhibitory interactions on the number of organizations. Fig. 4(B) demonstrates that the presence of toxins drastically reduces the number of viable organizations. Even when only three toxins with comparatively low producibility ($p_{\beta ,\text{produce}}=0.1$, their likelihood of being produced by a taxon) and sensitivity ($p_{\beta ,\text{sensitive}}=0.1$, their likelihood of being able to kill a taxon) are introduced, the expected number of organizations is roughly halved when considering just a single food resource. The effect of toxins becomes even more pronounced as the number of resources increases. For instance, in models with S=10 taxa interacting through M=30 different resources, the number of organizations in the absence of toxins is roughly ten times larger than in their presence. This arises because a larger number of resources implies higher specialization, which in turn leads to larger organizations that are more likely to be affected by inhibitory interactions.

Taken together, the number and size of organizations are shaped by both resource specialization and inhibitory interactions, which together determine the structural richness and variability of the extended hierarchy. Understanding these factors provides a foundation for exploring how ecological and interaction parameters control the potential coexistence patterns in microbial communities.

### 2.7 Influence of toxins on the extended hierarchy of organizations

Building on the previous analysis of factors shaping the number and size of organizations, we now focus on how inhibitory interactions affect the structure of the extended hierarchy. Toxins not only reduce the number of viable organizations but also alter the internal composition of interaction clusters and elementary organizations, thereby reshaping the pathways through which higher-order organizations can form. In this section, we investigate how the presence, number, and potency of toxins influence both the combinatorial diversity of organizations and the patterns of persistence within the hierarchy.

To quantify the effect of toxins on the extended hierarchy of organizations, we generated random models under various parameter settings (Fig. 4). For instance, a model with S=10 taxa, M=10 food resources and T=5 toxins, where each toxin is produced by a taxon with probability $p_{\beta ,\text{produce}}=0.1$ and a taxon is sensitive to it with probability $p_{\beta ,\text{sensitive}}=0.1$, contains on average 40 organizations (out of 1024 different taxa sets, black line). There are 50 non-persistent unions of these organizations on average (red line), which provide key information about the negative interactions mediated by toxins (see below).

To interpret these effects more mechanistically, taxa can be grouped according to their relationship with each toxin. Each toxin defines two disjoint groups: one containing all producers of the toxin, and the other group containing all taxa sensitive towards that toxin. A set of taxa can only constitute an organization if, for each toxin, the organization does not include members from both groups simultaneously.

Consequently, a union of organizations containing taxa from both groups of a toxin fails to be an organization, producing a red node in the extended hierarchy (Figs. 1(E) and 4). In other words, for a non-persistent union of organizations to occur, there must be at least one toxin-producing and one toxin-sensitive organization present in the union.

**Fig. 5:**
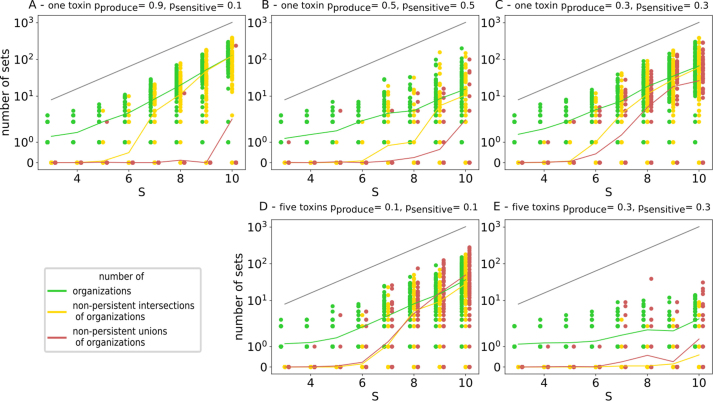
Number of organizations (green), non-persistent intersections of organizations (yellow), and non-persistent unions of organizations (red) in the extended hierarchy of 100 generated random models with M=10 food resources and parameters for the toxins as stated in the captions, considering one toxin (top) and five toxins (bottom), for different number of taxa S (x-axis). Solid lines depict the mean. Note that in panel A and panel B, each taxon is either sensitive to the toxin or producing it. Further, note that we also count the empty organization as an organization, thus, the minimum number of organizations is one. Vertical axis is symlog-scaled. (For interpretation of the references to colour in this figure legend, the reader is referred to the web version of this article.)

Interestingly, the number of toxin-producing and toxin-sensitive organizations increases with the abundance of neutral taxa, that is, taxa that neither produce a toxin nor are sensitive to it (cf. Fig. 5(C)–(E)). This occurs because toxin-producing and toxin-sensitive organizations can combine with organizations to form additional toxin-producing and toxin-sensitive organizations, respectively. As a result, the probability of forming non-persistent unions (red nodes) rises with the number of neutral taxa.

When considering a single toxin, individual hierarchies can contain many non-persistent unions, but these instances remain relatively rare (Fig. 5(A) and (B)). For a single toxin, the maximal number of non-persistent unions occurs when toxin producers, sensitive taxa, and neutral taxa are evenly distributed (data not shown). If neutral taxa are more abundant than this balance, the frequency of non-persistent unions decreases. This is because neutral organizations, which contain neither toxin producers nor sensitive taxa, are far more likely to be unable to form non-persistent unions, reducing the overall occurrence of red nodes in the extended hierarchy.

### 2.8 Measurement effort to reconstruct the hierarchy

In many practical settings, the underlying model of a microbial community is unknown, and the hierarchical structure of organizations must be inferred through measurements. A measurement is a set of taxa obtained from an experimental observation (e.g., 16S rRNA sequencing). The measured taxa sets form a hierarchy, displaying the subset relations of the measurements. An example of an incomplete extended hierarchy derived from real 16S rRNA measurements is given in a previous publication [25], illustrating the hierarchy construction from experimental datasets.

For the reconstruction, we assume that the population is in an attractor [25]. Because we know that only taxa forming an organization can persist (as discussed above), we further assume that a single measurement corresponds to an organization of the underlying, unknown model [25]. The effort to reconstruct the hierarchy is then quantified by the number of measurements required to recover the full set of organizations.

As intersections or unions of measurements may themselves be organizations, we term them additional intersections or additional unions, since they represent candidates for new organizations in the hierarchy.

A special case occurs when a set is simultaneously both a union and an intersection of measurements, which we term an additional organization. According to the model, such a set must indeed be an organization: each taxon is reproducible across the relevant intersections and superset measurements, demonstrating that coexistence is not hindered by toxins.

Additional intersections and unions provide valuable information for reconstructing the hierarchy:

• If an additional intersection or union is persistent, it indicates a previously unobserved organization.
• If it is non-persistent, it reveals constraints due to resource dependencies or toxins, thus providing insight into the community’s interaction structure.

To explore how measurement strategy affects reconstruction, we consider three types of experiments (detailed within Methods in the Simulating Measurements Section 4.3):

1. Newly encountered organization experiments: only previously unobserved taxa combinations are counted as new measurements.
2. Neutral encounter experiments: each organization has the same probability of being drawn with replacement.
3. Random encounter experiments: a random set of taxa is sampled, and the largest organization contained within that set is considered as the measurement.

In the presence of toxins, determining the largest contained organization is not always straightforward. Here, we assume that toxins act rapidly: any sensitive taxa are removed first, leaving a well-defined largest organization that reflects the immediate effect of inhibitory interactions.

#### 2.8.1 Measurement effort with respect to newly encountered organizations

To study how many measurements are required to recover each organization of the hierarchy - as at least additional union or an additional intersection - we assume in this section that organizations (measurements) are uniformly drawn without replacement. That is, only those measurements are considered that consist of a previously unobserved set of taxa. Repeated measurements based on the same taxa combinations can be neglected for the reconstruction of the hierarchy, as they cannot bring any change to the hierarchy.

**Fig. 6:**
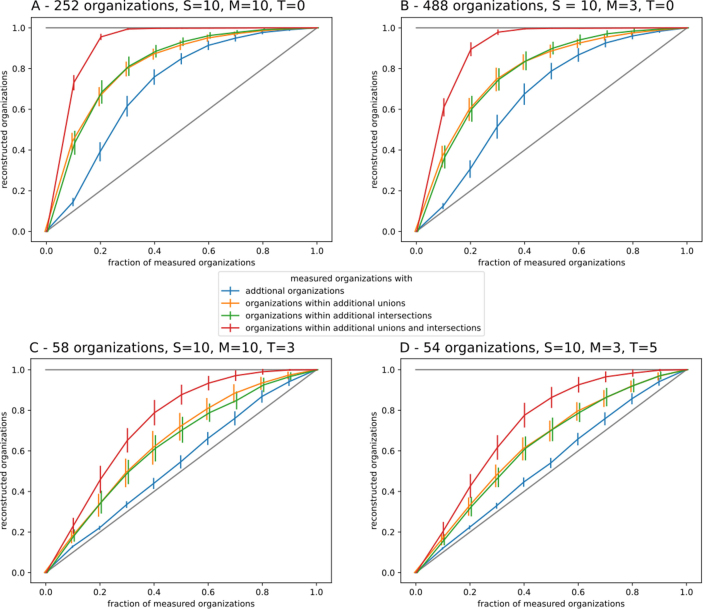
Fraction of reconstructed organizations based on the fraction of measured organizations. This is done on a random reaction network with S=10 taxa on M=10 food resources (left) and on M=3 food resources (right) with toxins (bottom) and without toxins (top). The presence of toxins hinders the reconstructability of organizations. If unions and intersections are included even small knowledge is sufficient to obtain huge parts of the complete hierarchy.

A set of taxa is identified as an organization if it appears either as a direct measurement or as an additional organization derived from the intersections and unions of previous measurements. Interestingly, the complete hierarchy can often be reconstructed without requiring all possible measurements (Fig. 6). In the absence of toxins, even a small fraction of measurements is sufficient to recover most of the final organizations.

Once all final organizations appear as additional intersections or additional unions, the search for new organizations can safely be restricted to these sets without loss of information. This highlights that the introduction of unions and intersections substantially enriches the reconstruction process (Fig. 6), as they provide new entry points for identifying previously unseen organizations. The contributions of additional unions and intersections are approximately equal, indicating a balanced role in extending the reconstructed hierarchy.

In contrast additional organizations - those simultaneously representing both a union and an intersection - require a larger number of measurements to emerge frequently. Their occurrence is limited to incomplete reconstructions, since they directly correspond to a valid measurement that has not yet been explicitly observed. A variation in the number of food resources (M) has only a minor influence on the reconstruction process when measurements are drawn without replacement (Fig. 6).

The presence of toxins, however, substantially alters this picture. Toxins reduce the likelihood that unions form new organizations because inhibitory interactions prevent certain combinations of taxa from coexisting. As a consequence, some additional intersections or unions may appear as potential organizations even though they are not truly persistent within the underlying system. This raises an important methodological question: how reliably can organizations be identified from measurements when toxins are present? In the following section, we therefore examine the false discovery rate, quantifying the fraction of falsely inferred organizations that emerge during the reconstruction of the hierarchy.

##### False Discovery Rate of Intersections and Unions

**Fig. 7:**
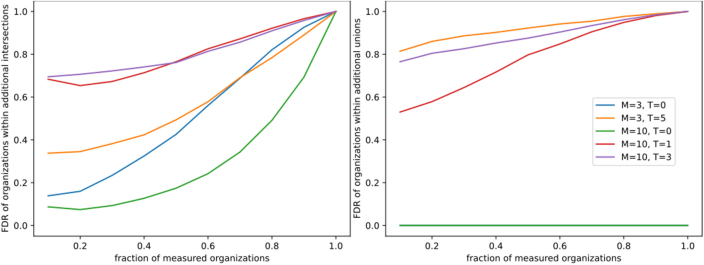
False discovery rate (FDR) of additional intersections (left) and additional unions (right) dependent on the fraction of measured organizations. In general FDR increases with the size of the reconstructed hierarchy. Without toxins (green and blue) additional unions are always new organizations. Under the presence of toxins most intersections and especially unions are not organizations. This is highly dependent on the internal reaction network structure. (For interpretation of the references to colour in this figure legend, the reader is referred to the web version of this article.)

Without expert knowledge, organizations within additional sets cannot be reliably identified. Therefore, we used the false discovery rate (FDR) to evaluate the accuracy of reconstructed organizations, which varies considerably with the structural properties of the underlying network (Fig. 7). The FDR was computed empirically as the fraction of falsely identified organizations among all predicted organizations, following the original definition by Benjamini and Hochberg [3]. It depends strongly on the intrinsic properties of the system, such as the composition of resources and toxins. Therefore, we tested several example hierarchies with varying resource-toxin compositions to assess the robustness of the observed effects (Fig. 7).

In the absence of coexistence-excluding interactions, additional unions always correspond to true organizations. However, even a single toxin increases the FDR for additional unions to about 0.5 (Fig. 7 right, red), meaning that only half of them represent valid organizations. This effect becomes stronger with more toxins (Fig. 7 right, yellow and violet).

Additional intersections can be either true or non-persistent intersections of organizations. Without inhibition, if less than one-third of the complete hierarchy is known, roughly four out of five additional intersections are true organizations (Fig. 7 left, blue and green). In contrast, the presence of toxins especially in combination with complex resource dependencies markedly increases the FDR (Fig. 7 left, red and violet).

In summary, additional unions can be interpreted as organizations only if inhibition is absent, whereas additional intersections-particularly small ones-should be treated with caution, as missing resource production may lead to loss of self-maintenance.

#### 2.8.2 Measurement effort for neutral encounter experiments

To estimate the actual number of measurements needed, we simulated neutral encounter experiments, in which measurements are drawn with replacement from the set of organizations. Because the same organization can be sampled multiple times, more measurements are generally required to reconstruct the full hierarchy. Still each organization is equally likely to be drawn as a measurement.

**Fig. 8:**
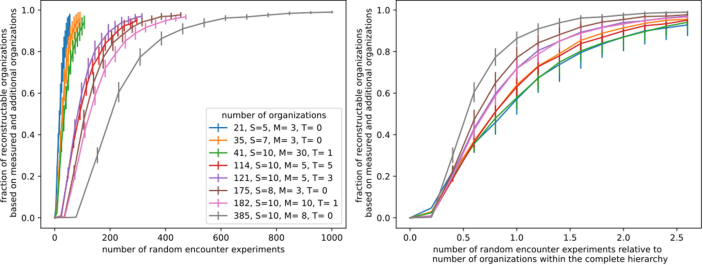
Measurement effort for neutral encounter experiments. The fraction of reconstructable organizations (measured and additional organizations) is shown as dependent on the number of measurements for different models. A label shows the total amount of organizations of that community along with the number of considered taxa S, food resources M, and possible toxins T. A taxon produces a toxin with probability $p_{\beta ,\text{produce}}=0.1$ and is sensitive to with probability $p_{\beta ,\text{sensitive}}=0.1$.

In unknown models, especially in the presence of toxins, the reconstruction relies primarily on directly measured organizations and additional organizations. The absolute number of required measurements depends mainly on the total number of organizations in the hierarchy (Fig. 8). Typically, roughly 2.5 times the number of organizations is sufficient to reconstruct over 80% of the hierarchy. Interestingly, systems with more organizations often require fewer measurements relative to their total (e.g., Fig. 8 right, gray and brown), reflecting a higher probability of recovering each organization early.

Toxins produce a similar effect as seen in smaller systems: the presence of inhibitory interactions reduces the likelihood of additional organizations (e.g., cf. Fig. 8 red and yellow or green and blue), so more organizations must be measured directly.

In summary, in neutral encounter experiments, at least two measurements per organization are generally sufficient to reconstruct ≈80% of the hierarchy, independent of the total number of organizations. Systems with a larger variety of organizations achieve this coverage even earlier, requiring only one measurement per organization to reach the 80% threshold.

#### 2.8.3 Measurement effort for random encounter experiments

**Fig. 9:**
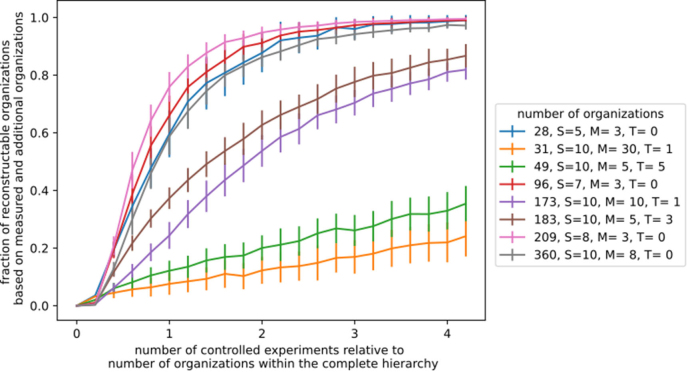
Measurement effort for random encounter experiments (organization generated from a random set of taxa). The fraction of re-constructable organizations (measured and additional organization) is shown as dependent on the number of random encounter experiments (relative to the total number of organizations). A label shows the total amount of organizations of that community along with the number of considered taxa S, food resources M, and possible toxins T. A taxon produces a toxin with probability $p_{\beta ,\text{produce}}=0.1$ and is sensitive to with probability $p_{\beta ,\text{sensitive}}=0.1$.

A more realistic measurement scenario can be simulated by starting from a random set of taxa and observing the resulting organization, referred to as random encounter experiments. In this setup, each organization can be reached from different numbers of initial taxa combinations, leading to a weighted probability of sampling each organization (Fig. 9). Consequently, some organizations appear more frequently in measurements, while others are rarer.

In systems without toxins, the number of measurements required relative to the total network size remains roughly unchanged. This indicates that the inferred organization weights do not substantially affect the overall recoverability of the hierarchy.

In contrast, in systems with toxins, recoverability is drastically reduced (Fig. 9). The inferred weights vary strongly between organizations: some become highly abundant in the measurements, while others are sampled very rarely. This reflects the selective effect of inhibitory interactions, which limits the range of coexisting taxa and biases the likelihood of encountering certain organizations.

### 2.9 Approach to reconstructing the model

A given hierarchy can be realized by multiple combinations of food resources and toxin interactions. Following the principle of Occam’s razor, the goal is to identify minimal models that explain the observed hierarchy, highlighting only the essential resource dependencies and inhibitory interactions.

Incomplete measurements often leave gaps in the reconstructed hierarchy. In communities without prior knowledge, these gaps can safely be filled only with additional organizations (sets that are simultaneously unions and intersections of measured organizations). By contrast, misinterpreting additional intersections as organizations can neglect dependencies on higher-level taxa, while misinterpreting additional unions can overlook inhibitory interactions that prevent coexistence. Similarly, ignoring small organizations may overestimate self-maintenance complexity, and missing larger organizations may falsely suggest barriers to coexistence. Therefore, additional unions should only be considered as non-persistent if there is substantial evidence that the majority of major organizations have been measured.

In the following, we sketch the path towards two algorithms that build on the methods described here to infer the minimal sets of required toxins and essential food resource interactions from a measured hierarchy. A full formal implementation and evaluation will be developed in a forthcoming work.

#### 2.9.1 Towards Inferring Minimal Sets of Toxins

Assuming a measured hierarchy of organizations, the minimal sets of toxins required to explain the hierarchy can be inferred from the non-persistent unions, as sketched in the following. The procedure identifies minimal pairs of taxa sets that are unable to coexist, termed taxa impairings. These taxa impairings are two minimal sets of taxa that do not occur together in any organization (measurement). The procedure then determines a minimal set of taxa impairings needed to explain all non-persistent unions. Each set of these minimal impairings can then be translated into a toxin table alternative, and the smallest toxin tables represent parsimonious explanations for the observed non-persistence.

In the example (Fig. 1(E)), there are two non-persistent unions, {a,b,c,d} and {a,b,c,d,e}. For the union {a,b,c,d}, the connected subsets {a,b,d} and {b,c,d} reveal that taxa a and c cannot coexist. The resulting taxa impairing ({a},{c}), also explaining the second union, is consistent across the hierarchy, because there is no organization containing both a and c. As a result, there are two minimal toxin tables containing a single toxin produced by one of the taxa (a or c) to which the other is sensitive (Table 2, $t_{1}$).

**Table 2:** Obtained possible food resource and toxin table fillings of the extended hierarchy of Fig. 1(E) based on the described methods. The markers +, producing, −, needing/sensitive to and 0, unaffected, in black represent the actual filling as in the extended hierarchy. The retrieved alternatives are marked in purple.

#### 2.9.2 Towards inferring minimal sets of required food resources

Having identified the minimal sets of toxins required to explain non-persistent unions, the next step would be to infer the minimal set of resources and cross-feeding interactions that explain all measured organizations and all non-persistent intersections of these organizations.

In principle, it is possible to enumerate all possible food tables to explain a measured hierarchy. However, this is computationally infeasible for larger systems. Therefore, in the following, we suggest a heuristics that determines how many resources each taxon requires and which taxa can potentially supply them. This allows us to construct a food table step by step using the following heuristic procedure:

The procedure starts from an empty food table, which implies a maximal hierarchy of organizations, containing more organizations than actually measured. Iteratively, the table is adjusted until the constructed model implies precisely all measured organizations. There are two possible adjustments:

1. Introducing a new resource and adding a requirement for this resource to a taxon, that is, adding “−” in the food table. This adjustment leads to a reduction in the organizations implied by the food table.
2. Adding productions of existing resources, that is, adding one or more “+” in the food table. This will increase the number of organizations implied by the food table.

Among all possible adjustments, minimal solutions are selected to keep the number of resources low while reproducing the measured hierarchy with increasing accuracy.

As an example, the hierarchy within Fig. 1(E) contains six organizations (measurements): {a},{a,b,d}, {a,b,e},{b,c,d},{a,b,d,e}, and {b,c,d,e}. Given that a and c cannot coexist (due to toxins), an initial empty food table implies 29 organizations, that is, 23 too many. By assigning each taxon apart from a a separate required resource, four resources suffice to eliminate the smallest extra organizations ({b},{c},{d},{e}). Next, context-dependent interaction clusters, such as {b,e} and {b,d}, are formed by allocating resources produced by co-occurring taxa, while the context-free cluster {b,c,d} has to set up a new cross-feeding cycle involving all three taxa. Through careful allocation, all measured organizations are explained without extra ones, and the total number of resources can be further minimized by combining resources shared among multiple taxa. In this example, the final minimal food table (Table 2) requires the same amount of resources like the “ground truth” (Fig. 1(A)) used to generate the measurements. However, the detailed cross-feeding relationships deviate, that is, a can produce only one resource and for b two resources can be sufficient.

## 3 Discussion and conclusion

Assuming that community composition is not primarily driven by stochastic immigration or extinction events, it can be understood through the network of interactions among its members. In microbial ecosystems, such interactions are commonly captured by Lotka-Voltera models, focusing on direct taxa interactions, or consumer-resource models, where taxa influence one another indirectly through the uptake and release of shared metabolites. Within this mechanistic framework, the application of Chemical Organization Theory (COT) provides a structural and parameter-independent way to identify all self-sustaining combinations of taxa - termed organizations - and thereby reveals the complete hierarchical structure of coexistence. This framework connects to classical ecological concepts such as niche overlap, feasibility domains, and community assembly rules, while offering a formal, systems-level representation of their organizational basis.

Without knowledge of exact stoichiometric coefficients, every combination of persistent states is also persistent. To incorporate coexistence-excluding negative interactions, COT was extended to include inhibitors, here referred to as toxins.

The central assumption is that each taxon requires all specified resources for self-replication, whereas the presence of a single toxin to which it is sensitive is sufficient to prevent its persistence.

The derivation of taxa-level reactions requires identifying the minimal sets of producers supplying the required resources for each taxon. As these are essential rather than substitutable resources, the problem can be formulated as a minimal hitting set problem, which is NP-hard. This procedure parallels the reformulation of consumer-resource models with essential resources into generalized Lotka-Volterra models that also account for group-wise (higher-order) interactions.

The resulting hierarchical structure defined by the set of organizations, can be extended by including non-persistent intersections and non-persistent unions. This extended hierarchy is particularly informative when considering experimental reconstruction from measured community compositions. Our results indicate that intersections and unions of the already measured taxa sets hold critical information about underlying interactions. Controlled experiments targeting these sets would therefore be especially valuable for model validation.

The introduction of toxins expands the number of representable hierarchies but also imposes structural constraints: since toxins cannot be neutralized, only hierarchies are realizable in which all supersets of non-persistent unions are also non-persistent. Any empirical observation contradicting this condition indicates the need to relax these assumptions and adopt a more general model formulation.

Each organization within the hierarchy is composed of interaction clusters-the fundamental building blocks of coexistence. These clusters comprise taxa that are mutually dependent and cannot coexist in any smaller subset. Non-persistent intersections between organizations expose shared structural elements, illustrating how interaction clusters are interlinked throughout the hierarchy.

Across simulated models, we found that both toxins and the number of food resources influence the number of possible organizations. A large number of resources corresponds to a high degree of specialization, which reduces the likelihood of forming persistent multi-species communities. Thus, while increased specialization can stabilize certain interactions, it also narrows the feasible coexistence space - consistent with predictions from modern coexistence theory.

When the underlying model is unknown, the hierarchy can be reconstructed empirically by measuring stable taxa combinations, each representing an organization. The reconstruction can be extended by additional unions and intersections that are potential organizations. Unions without toxins can be safely accepted as organizations, whereas in toxin-containing systems, such unions or intersections must be experimentally verified to avoid false positives. The false discovery rate (FDR) rises sharply when inhibitory interactions are present, underscoring the need for targeted experiments focusing on uncertain or underrepresented combinations. Simulations of random encounter experiments confirmed this bias, showing that in systems with toxins, certain organizations are sampled far more frequently than others, while parts of the hierarchy remain unexplored.

Because an extended hierarchy can arise from multiple alternative models, a minimal explanatory model is preferred, highlighting only essential resource dependencies and inhibitory interactions. Once a complete extended hierarchy is obtained, the minimal number of required toxins and food resources can be inferred.

Our approach, while general, makes several simplifying assumptions: each taxon requires exactly the listed resources; one toxin is sufficient to prevent coexistence; food resources and toxins are distinct entities; stoichiometry, spatial structure, and quantitative competition are neglected; and toxins cannot be neutralized. However, the strength of this framework lies in its extensibility. Each of these assumptions can be systematically relaxed, as COT is defined for arbitrary reaction networks and naturally extends to compartmentalized or spatially continuous systems. Future work may therefore incorporate stoichiometric constraints, metabolic subnetworks, or localized interactions. Further extensions might incorporate stochastic dynamics [21] and spatial heterogeneity [23] by using stochastic and distributed organizations, respectively.

A particular challenge remains the treatment of negative interactions: while the current approach handles them in a minimal form, generalizing this treatment to complex, nonlinear systems will be an important direction for further research.

## 4 Methods

### 4.1 Random model generation

Random consumer-resource-toxin models are generated to study the effect of inhibitory interactions on the hierarchical structure of microbial communities. A random model is constructed by filling the food and toxin table (Fig. 1(A) randomly in the following way:

• Food table: For each taxon i and resource α, the entry is randomly assigned + (produced), − (required), or 0 (neither) with equal probability $p_{\alpha ,\text{produce}}=p_{\alpha ,\text{sensitive}}=1/3$
• Toxin table: For each taxon i and toxin β, the entry is randomly assigned + (produced), − (sensitive), or 0 (neither). The probability of + is $p_{\beta ,\text{produce}}$, and the probability of − is $p_{\beta ,\text{sensitive}}$.

#### 4.1.1 Transformation of a Model to a Reaction Network

To analyze the dynamics of a consumer-resource-toxin (CRT) model, each model is transformed into a reaction network. For each taxon i∈{1,…,S}, the set of required resources is defined as $\delta_{i}=\{\alpha |\rho_{i,\alpha }=-1\}$. This yields a replicator reaction for each taxon: $i+\sum_{j\in \delta_{i}}j\to 2i$. The replicator reactions are extended with:

1. A general outflow for each taxon: i→∅
2. An outflow for each toxin $j\in \eta_{i}$ to which taxon i is sensitive: i+j→j.

To express reactions solely in terms of taxa, we identify minimal sets of taxa $S^{\prime }\subseteq \{1,\ldots ,S\}$ that produce all resources required by taxon i, i.e., $\delta_{i}\subseteq \underset{j\in S^{\prime }}{⋃}\eta_{j}$, with no proper subset of $S^{\prime }$ covering $\delta_{i}$. This task is equivalent to the minimal hitting sets problem and is therefore NP-hard.

Problem formulation: Given a set of taxa S={1,…,S} and a set of food resources R={1,…,R}. Each taxon i∈S is defined by its produced resources $\epsilon_{i}$. For a taxon $s^{*}$ with required resources $\delta_{s^{*}}\subseteq R$, find all minimal subsets $S^{\prime }\subseteq S$ such that $\delta_{s^{*}}\subseteq \underset{i\in S^{\prime }}{⋃}\epsilon_{i}$, and no subset $S^{\prime \prime }\subset S^{\prime }$ satisfies the same.

Theorem 1 *Finding all such minimal subsets* $S^{\prime }$ *is NP-hard.*

Proof A candidate solution $S^{\prime }$ can be checked in polynomial time.

To show NP-hardness, the minimal hitting sets problem can be reduced to this task: each vertex corresponds to a taxon, and each hyper-edge corresponds to a resource. Finding all minimal subsets producing all required resources is equivalent to solving the minimal hitting sets problem, which is NP-hard.  □

Although the reduction of resource-taxa interactions to direct taxa interactions is NP-hard, practical inference can be achieved by using heuristic strategies. A simple greedy approach, for instance, iteratively selects the taxon or resource that resolves the largest number of remaining dependencies, analogous to successively adding the most broadly beneficial species or metabolite until all requirements in the community are met. Such approximations, possibly combined with stochastic selection among equally beneficial candidates, provide biologically interpretable and computationally efficient routes to infer near-minimal sets even in large systems.

Since a taxon can be replicated by multiple minimal sets of producers, each taxon may have multiple replication reactions corresponding to these alternative sets. Similarly, each toxin-specific reaction is instantiated for each producer taxon. This completes the transformation of the CRT model into a reaction network suitable for subsequent analysis using Chemical Organization Theory.

### 4.2 Generating the extended lattice

To infer an organization from the reaction network, a set of taxa must be closed and self-maintaining. Closure is trivially satisfied, as only taxa already in the set are considered for replication. Self-maintenance requires that all necessary resources for each taxon are produced within the set. To incorporate toxins, a set can only qualify as an organization if no taxon is sensitive to any toxin produced by members of the set.

To generate all organizations, the previously identified minimal producing sets for each taxon are first extended to include the resource needs of all taxa within those sets. These minimal combinations are then further extended by their unions to construct the lattice of organizations without toxins. The extended lattice accounting for toxins is obtained by removing all organizations in which at least one taxon is sensitive to a toxin produced by the set. Finally, intersections and unions of all organizations are incorporated to produce the complete extended lattice.

### 4.3 Simulating measurements

Given a model, we simulate measurements using three approaches:

1. Newly encountered organization experiments. Only taxa combinations not previously observed are counted as new measurements. This approach mimics an idealized scenario where each measurement provides completely novel information.
2. Neutral encounter experiments. Organizations are drawn uniformly at random with replacement. Each organization has the same probability of being measured, independently of previous measurements. This reflects experiments where the initial composition does not bias the resulting community.
3. Random encounter experiments. A community is initialized with a random set of taxa: each taxon is included independently with probability p=0.5. The resulting measurement is the organization generated by this set. That is, taxa affected by toxins produced within the set are removed, followed by iterative removal of taxa whose required food resources are not supplied by the remaining taxa. The largest organization contained within the initial set is then recorded as the measurement. This procedure is analogous to triadic percolation [31].

## CRediT authorship contribution statement

**Beatrice Ruth:** Writing – review & editing, Visualization, Validation, Software, Resources, Methodology, Formal analysis, Data curation **Bashar Ibrahim:** Writing – review & editing, Supervision, Methodology, Formal analysis, Conceptualization. **Peter Dittrich:** Writing – review & editing, Supervision, Funding acquisition, Formal analysis, Conceptualization.

## Declaration of competing interest

The authors declare that they have no known competing financial interests or personal relationships that could have appeared to influence the work reported in this paper.

## Data Availability

Data for this study can be generated from the repository at https://git.uni-jena.de/ne78xoy/crminterfer. For any inquiries, please contact ibrahim.b@gust.edu.kw or peter.dittrich@uni-jena.de.
